# sim1000G: a user-friendly genetic variant simulator in *R* for unrelated individuals and family-based designs

**DOI:** 10.1186/s12859-019-2611-1

**Published:** 2019-01-15

**Authors:** Apostolos Dimitromanolakis, Jingxiong Xu, Agnieszka Krol, Laurent Briollais

**Affiliations:** 1 Lunenfeld-Tanenbaum Research Institute, Mount Sinai Hospital, 60, Murray Street, Toronto, ON M5T 3L9 Canada; 2 Dalla Lana School of Public Health, University of Toronto, Toronto, M5T 3L9 Canada; 3 Department of Statistical Sciences, University of Toronto, Toronto, M5S 3G3 Canada

**Keywords:** Simulation, Sequencing, NGS, 1000 genomes, Linkage disequilibrium, Pedigree data

## Abstract

**Background:**

Simulation of genetic variants data is frequently required for the evaluation of statistical methods in the fields of human and animal genetics. Although a number of high-quality genetic simulators have been developed, many of them require advanced knowledge in population genetics or in computation to be used effectively. In addition, generating simulated data in the context of family-based studies demands sophisticated methods and advanced computer programming.

**Results:**

To address these issues, we propose a new user-friendly and integrated *R* package, *sim1000G*, which simulates variants in genomic regions among unrelated individuals or among families. The only input needed is a raw phased Variant Call Format (VCF) file. Haplotypes are extracted to compute linkage disequilibrium (LD) in the simulated genomic regions and for the generation of new genotype data among unrelated individuals. The covariance across variants is used to preserve the LD structure of the original population. Pedigrees of arbitrary sizes are generated by modeling recombination events with *sim1000G*. To illustrate the application of *sim1000G,* various scenarios are presented assuming unrelated individuals from a single population or two distinct populations, or alternatively for three-generation pedigree data. *Sim1000G* can capture allele frequency diversity, short and long-range linkage disequilibrium (LD) patterns and subtle population differences in LD structure without the need of any tuning parameters.

**Conclusion:**

*Sim1000G* fills a gap in the vast area of genetic variants simulators by its simplicity and independence from external tools. Currently, it is one of the few simulation packages completely integrated into *R* and able to simulate multiple genetic variants among unrelated individuals and within families. Its implementation will facilitate the application and development of computational methods for association studies with both rare and common variants.

**Electronic supplementary material:**

The online version of this article (10.1186/s12859-019-2611-1) contains supplementary material, which is available to authorized users.

## Background

With the emergence of next-generation sequencing (NGS) technologies, the amount of genetic data generated every year grows exponentially. Developing new methods for analyzing these data is an area of active research, not only in human populations but also in plant and animal species. It is a common practice to generate large number of simulated datasets for validation and comparison of novel bioinformatics tools and statistical methods.

Simulation programs are a key component of genetic and genomic research, useful for improving our understanding of the mechanisms underlying complex biological processes [1]. Unlike experimental data, where the “truth” is unknown, simulated data sets are created under particular scenarios to mimick real biological systems. These so-called in silico data sets can be used for example, to assess different hypotheses, validate statistical methods and compare the power of different analytical methods. Simulations can also be used to evaluate conditions such as evolutionary history, which gives rise to existing genomic data [2]. Genetic simulators might also be useful for creating some generalizable benchmark data sets and/or reference simulation program(s) for the user community [3]. These benchmark data sets could include, for example, multi-ethnic sequence data files. Currently, there is a plethora of simulation programs and packages but a lack of established criteria for their evaluation. Therefore, it is difficult for investigators less familiar with simulation methodologies to select an appropriate simulation program that satisfies their needs. In many instances, researchers have to develop customized softwares for the simulation of genetic data. Chen et al. [2] reviewed several issues of the journal “*Genetic Epidemiology*” and found out that out of 36 articles that included simulated genetic data, only 8 of them used genetic simulators or simulators already catalogued in the Genetic Simulation Resources page of NIH [4]. Many resources used for the development and implementation of genetic simulators are likely redundant.

While many approaches have been introduced for simulations of genetic variants data [5] such as *HapGen2* [6], *simuPOP* [7] and *simuRARE* [8], many of those rely on population evolution theory and pose additional complexity to researchers developing methods in statistical genetics.

To address some of the shortcomings of existing genetic simulators, we implemented a genetic variants simulator, *sim1000G*, which is user-friendly, completely integrated into *R* and fits various simulation purposes. Implementing *sim1000G* in *R* also facilitates the integration of analytic methods for genetic association tests, many of them being also available in *R*. S*im1000G* has been designed to simulate existing genetic variants with a wide range of MAFs under different study designs including independent individuals and pedigree data. It was not developed to simulate sequence data nor novel genetic variants. Other types of genetic variants such as MNPs, indels, CNVs, functional annotations can be simulated if they are included in the input VCF file, as long as they are biallelic. The impact of evolutionary processes (e.g. natural selection on one or more mutations with its impact on surrounding variants) and the impact of demographic models (e.g. admixture populations) was not considered either although a easy way to simulate admixed populations is given as illustration (see Results section). The value of *sim1000G* is also its ability to create benchmark genetic variants data sets that can be used subsequently for assessing genetic association methodologies. Compared to most genetic simulators, the integration of our genetic simulator into *R* allows a much simpler workflow and minimal set-up time for the users of the package. The capacities of *sim1000G* allow the simulation of genotype data among unrelated individuals as well as within pedigrees of arbitrary sizes, a feature absent from most existing genetic simulators.

## Implementation

### Simulation of genetic variants among unrelated individuals

**Fig. 1:**
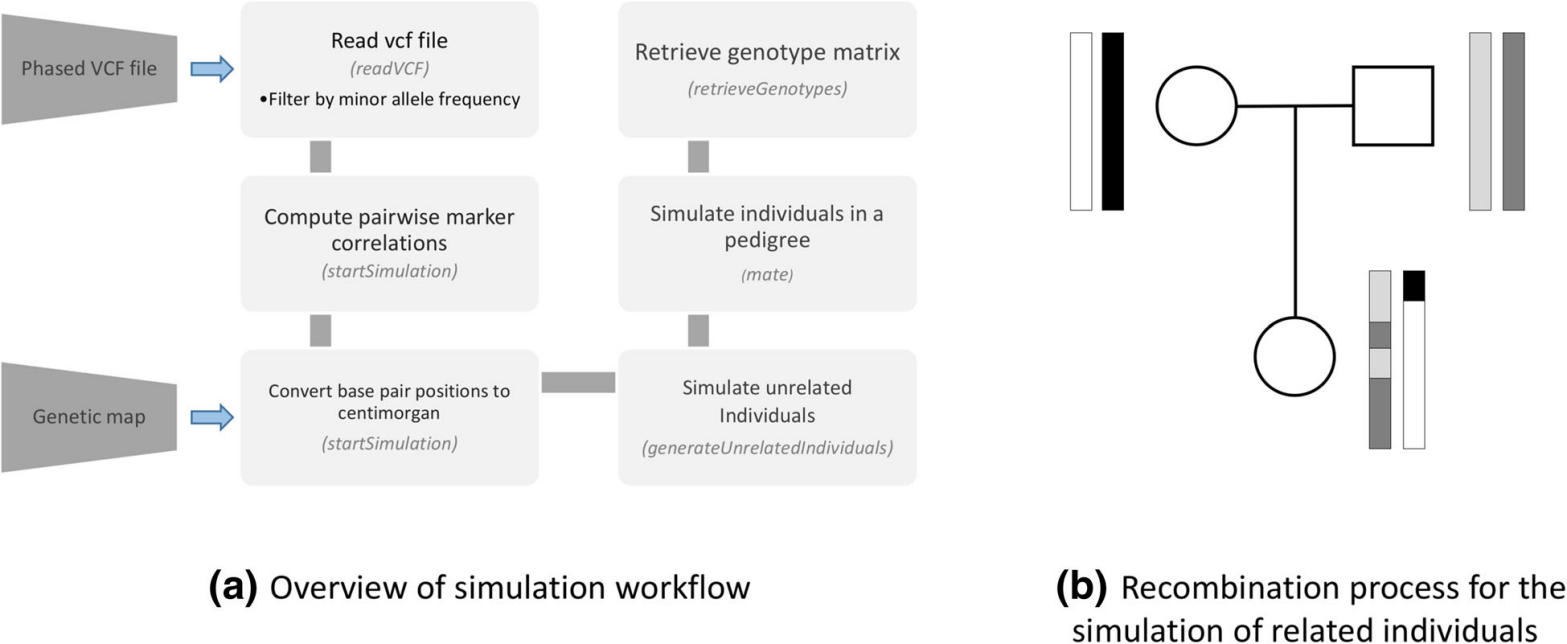
**a** Overview of our simulation workflow (function names in parenthesis). **b** Generating related individuals in sim1000G by following recombination events

A realistic genetic simulator of genetic variants data should preserve the minor allele frequencies (MAF) distribution and the LD patterns observed in a single homogeneous population or in an admixed population. With *sim1000G*, this is achieved by reading a phased VCF file containing both rare and common genetic variants located on a specific chromosomal region and for a given population. A common source of VCF inputs is the phased variant calls from Phase III 1000 genomes sequencing data [9], where variant calls are available on 2504 samples across 26 populations. For example, the initial population could be the European descent subset of the 1000 Genomes and the gene region be SMAD5. Generating VCF files for *sim1000G* can be performed using the *bcftools* package [10]. An overview of the simulation workflow is shown in Fig. 1.

There is no current limit on the size of the simulated genomic region, i.e. up to a whole chromosome in length, but the number of genetic variants should not exceed the computational capability of the *R* environment. There is an option in *sim1000G* for filtering genetic variants with respect to a minimum or maximum MAF and for the number of variants allowed, to speed-up computations. It is also possible to simulate only functional variants or specific classes of variants by providing a VCF file matching the required conditions.

After reading through the VCF file, the haplotypes for a particular population are extracted and the correlation between each pair of genetic variants is estimated. The *hapsim* package in *R* [11] is used for this step, wherein a haplotype is modeled as a multivariate random variable and the corresponding marginal distributions and pairwise correlation coefficients are estimated. *Hapsim* provides a computationally efficient algorithm to generate large pools of haplotypes, which are then integrated into *sim1000G*. The computation time for this step is proportional to the square of the number of genetic variants and is the most time-consuming step when *sim1000G* is applied to regions with large number of variants. The size of the region in Mb does not affect the simulation speed.

Simulating genetic variants for a new individual requires the generation of two simulated haplotypes for the genomic region considered using *hapsim* functionality. This step ensures that the simulated variants data capture both the allele frequency distribution, short and long-range LD structure from the genome as well as recombination hotspots. To enable higher computational efficiency, large pools of haplotypes are computed in batches. Each time a VCF file is read, a pool of 1000 haplotypes is automatically generated. Once this pool is exhausted, another pool of 1000 haplotypes is generated. The generation of unrelated individuals is performed with the function *generateUnrelatedIndividuals* in *sim1000G*.

With this feature, *sim1000G* can simulate genomic regions with a wide range of sizes and up to a full chromosome. Multiple chromosomes can also be simulated as long as the corresponding VCF files are provided. Examples of VCF files from across the genome are provided on the *sim1000G* github page.

The computer memory and computational burden grow in proportion to the square of number of variants considered. Simulations of up to 1000–2000 variants can be performed easily on a laptop computer while the generation of 4000 to 10,000 variants requires the use a workstation computer with sufficient memory.

### Simulation of genetic variants among pedigrees

Since many genetic analyses use pedigree data including linkage or family-based association studies, an important feature of *sim1000G* is its ability to simulate genetic variants among family relatives. A set of functions in *sim1000G* allows the modeling of recombination events and offspring formation assuming diploid individuals and autosomal chromosomes. This functionality can be used to simulate pedigrees of arbitrary sizes together with realistic genetic variants datasets that preserve the LD structure of the genomic region considered.

For this purpose, a detailed genetic map of the simulated region is needed. For human autosomal data, the genetic maps can be downloaded automatically from an online database on github (https://github.com/adimitromanolakis/geneticMap-GRCh37). We provided detailed genetic maps for all chromosomes obtained by re-mapping (lifting) the coordinates of the HapMap Phase II genetic map from build 35 to GRCh37. The original map was generated as part of the HapMap project [12]. Locations in centimorgans of each variant included in the simulation are computed from the corresponding base pair position.

Modeling recombination events with *sim1000G* is performed by selecting one of two models: an interference chi-squared model or a simple no-interference model. These models are used to generate inter-recombination distances on a chromosome and the recombination events that occur in the simulated genomic region are used to recombine the parental haplotypes. The model with interference was adapted from a two-pathway model previously described in Housworth and Stahl [13].

The function *mate* automates the above process and generates one or more offspring as specified in the pedigree structure from two previously simulated individuals.

Analysis methods dealing with familial data often require the estimation of identity by descent (IBD) probabilities between pairs of relatives. Through its simulation model, *sim1000G* tracks all ancestral haplotypes and alleles for each recombination event. This allows the computation of the exact IBD state at each position of the simulated region. The function *computePairIBD12* computes the exact IBD 1 and IBD 2 proportions for each pair of individuals.

### Computational efficiency

**Fig. 2:**
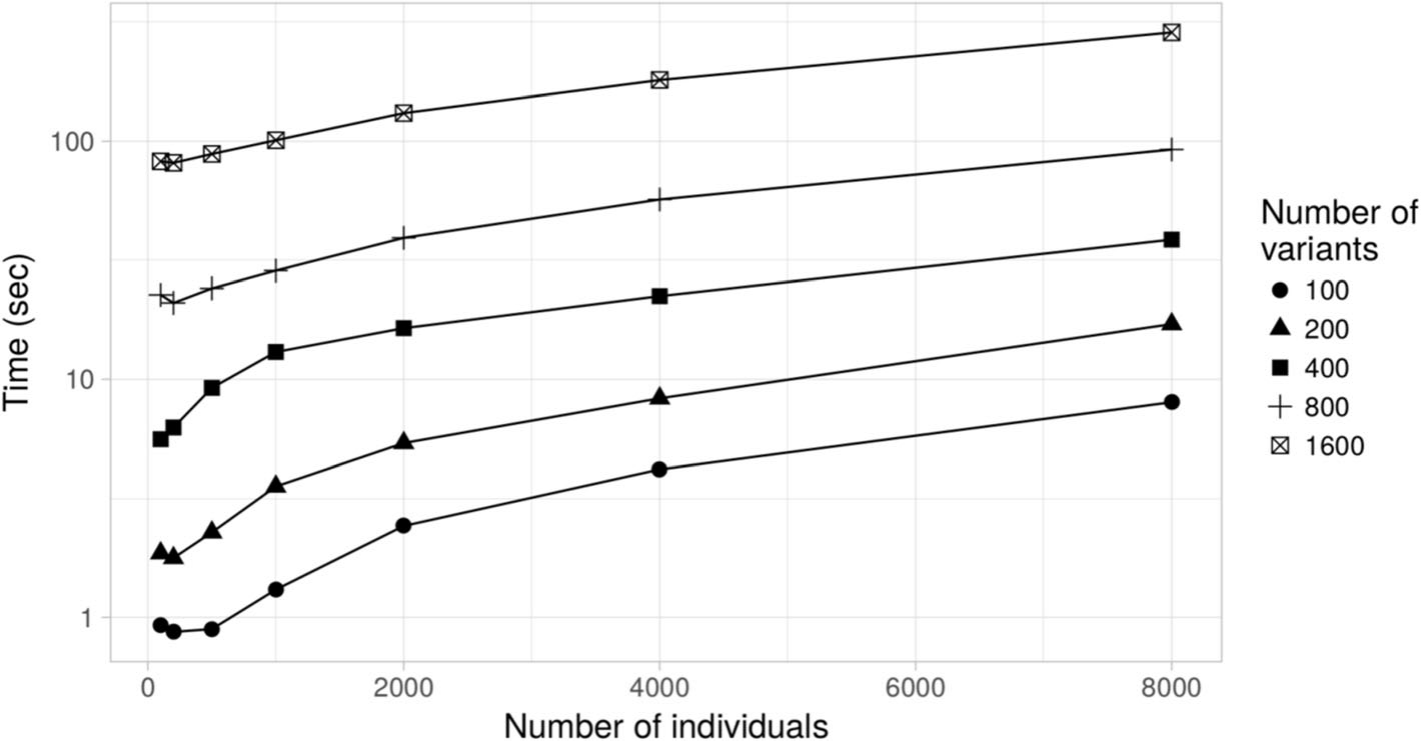
Running time of sim1000G when simulating a specific number of individuals and number of variants (timings include the simulation initialization time). The simulated region length does not affect the simulation time

The total running time of *sim1000G* was evaluated using a laptop computer with a 2GHz processor and 4GB of RAM. Only one CPU core, out of the 4 available, was used for all timing reports. The number of simulated individuals varied from 100 to 8000 and the number of variants from 100 to 1600. Even when considering thousands of individuals, the entire simulation process with *sim1000G* was completed in less than 10 s. The simulation of an entire genomic region of size 1MBp with 400 variants on 4000 individuals was finished in less than 8 s (Fig. 2), including the initialization time of the simulator.

## Results

### Comparison with other genetic variants simulators

We compared *sim1000G* to two other competing and well established genetic variants simulators: *hapgen2* [6] and *simuGWAS* [14]. We assessed how these simulators preserve the allele frequency distribution and correlation structure across genetic variants in a real genomic region. We used the default parameters specified by each software (Additional file 1) and simulated 2000 genetic variants spanning location 1 to 10MBp on chromosome 1.

#### Comparison of allele frequency distribution

**Fig. 3:**
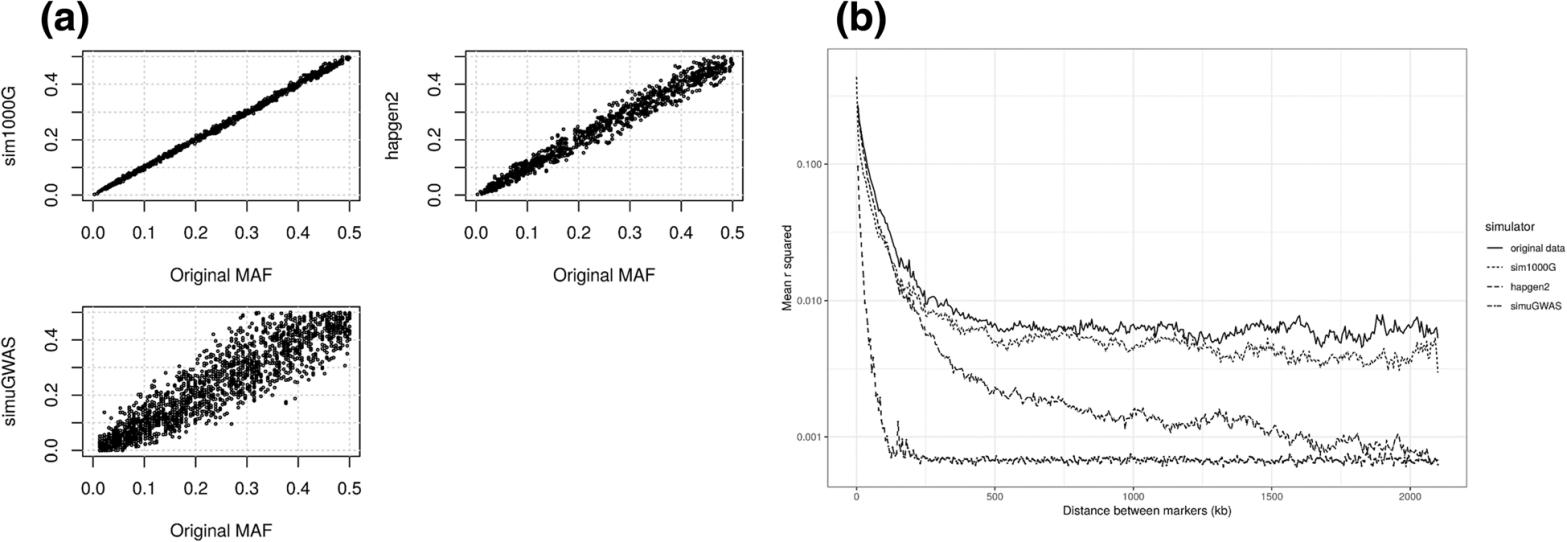
Comparison of simulated genetic variants to their original population. **a**: Allele frequency comparison between the original genetic variants and the simulated ones. **b**: Decay of LD patterns for the original data and the 3 simulators tested. Each curve shows the average value of pairwise LD (r^2^) between genetic variants with respect to the distance between these variants

Among the three simulators tested, *sim1000G* provided the most accurate allele frequency distribution estimates compared to the original data (Fig. 3a). The simulator *hapgen2* seems to slightly underestimate the MAF and has larger variability than *sim1000G.* Finally, *simuGWAS* has excessive variability and the allele frequency estimation diverged widely from the original data. This latter software does not aim to preserve MAF distributions as best as possible because the seed population and resulting population are both “samples” of the truth.

#### Comparison of correlation structure

The LD structure across genetic variants was the most accurately estimated with the simulators *sim1000G* and *hapgen2*, which preserved both long and short range LD (Fig. 3b). *Hapgen2* yielded better LD estimates for pairs of genetic variants distant less than 250 kb apart while *sim1000G* performed better to capture subtle patterns of long-range LD for pairs of variants more than 250 kb away from each other. The simulator *simuGWAS* could not estimate accurately the patterns of LD observed in the original population.

### Correction for population structure in genetic association studies

Population stratification is a common problem in genetic association studies, usually arising when cases and controls are sampled at differential rates from genetically divergent populations [15]. Methods based on principal components analysis can be applied to correct the test procedure for type-I error inflation [16]. In this context, being able to simulate realistic data sets mimicking population stratification problems can help evaluating how particular methods correct this bias. Our new simulator *sim1000G* has this capacity. As an illustration, we generated datasets of genetic variants from different ethnic groups. The population genetic heterogeneity leads to a significant number of false positive associated variants. We obtained the *p*-values for the association tests based on the SKAT method [17], before and after correction for population stratification.

We extracted a set of 200 genes from the 1000 genomes sequence data located on chromosome 4. Individuals from two distinct populations were selected: a European subset (populations CEU, TSI and GBR) and an African subset (populations ASW, LWK and YRI). For each gene, we filtered out genetic variants with MAF < 2% and generated the corresponding VCF files for use with *sim1000G*.

In total, 1000 replicate datasets were generated for each gene, with a total of 2000 individuals in each replicate. To create distinct LD patterns and allele frequency distributions, each ethnic group was generated independently with *sim1000G* and the genotypes were combined to create common sets of variants.

An outcome *y*_*i*_ was generated for each individual *i*, given its simulated genotypes *G*_*i*, *j*_, where *j* ∈ {1,  … , *J*} denotes the genetic variant *j* from a standard logistic regression model:

1 $$ logit\left(P\left\{{y}_i=1\right\}\right)={b}_0+{b}_1{s}_i+{\sum}_{j=1,\dots, J}{G}_{i,j}\ast {c}_j, $$

where b_0_ is a baseline parameter, s_i_ a population stratification term, assumed 0 for individuals from the European subset and 1 otherwise, b_1_ is the odds ratio of the disease risk between Africans and Europeans, and c_j_ the effect size (i.e. log odds-ratio) for a specific variant *j* (j = 1,…J).

Each simulated dataset included 3 causal genes, each with J = 10 causal genetic variants. The effect size of each variant was fixed in the range of log(1.5) to log(5). The number of individuals from African descent varied between 0 to 400 in order to simulate different magnitudes of population stratification effects.

SKAT [17] was used to obtain the association test *p*-value under 2 scenarios: (a) with no covariates or (b) with the ethnic group as covariate as a way to adjust for population stratification.

**Fig. 4:**
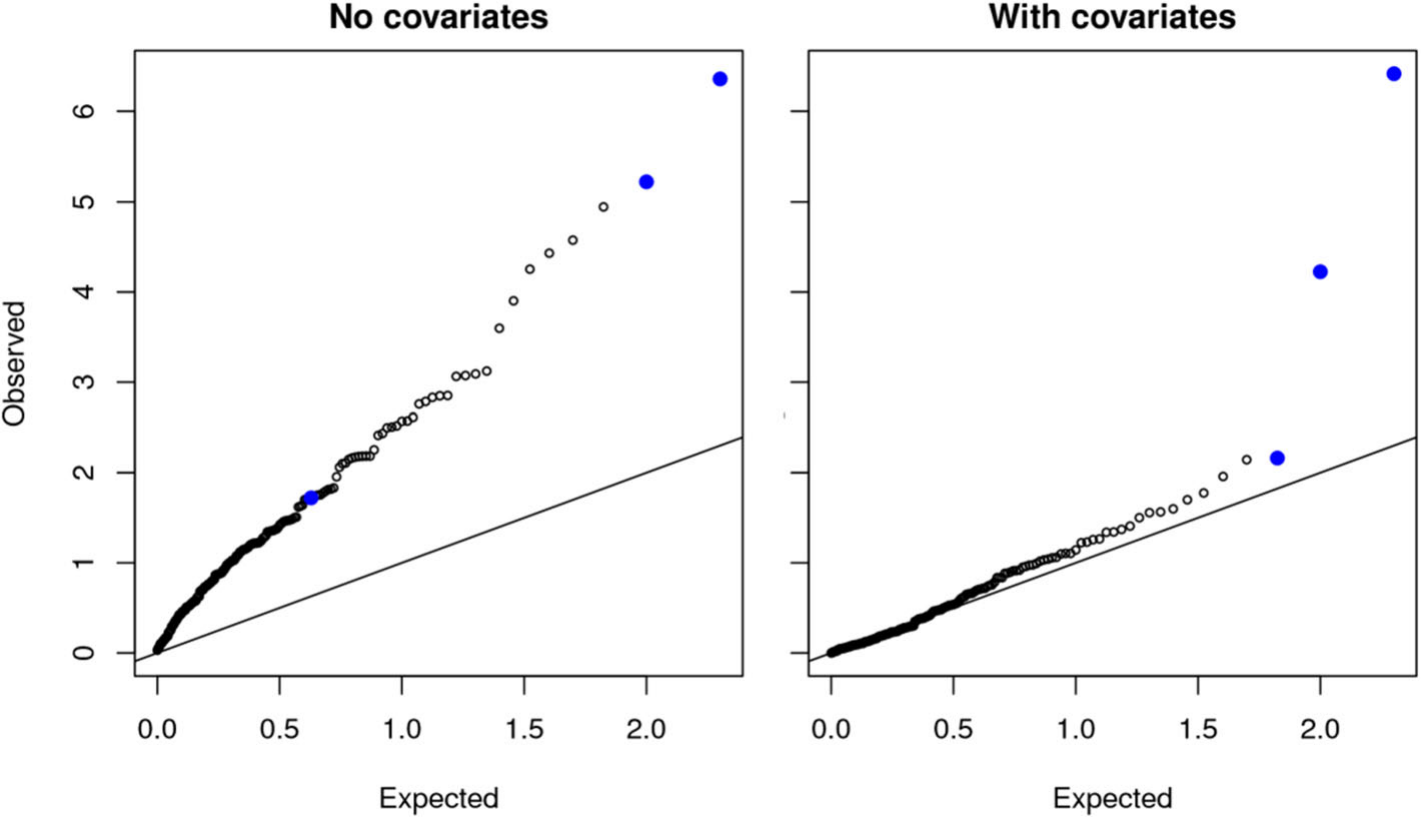
Examples of 2 Q-Q plots of SKAT *p*-values under two simulation scenarios: **a** Significant population stratification and no covariates adjustment, (**b**) With covariate adjustment. Solid points: true causal genes. Total number of genes was 200 with 3 causal genes, each with 10 causal variants

The results from 16 different simulation scenarios are shown in Table 1. In Fig. 4, an example of a QQ-plot representing the simulation results is given. The SKAT test is able to correct for population structure bias with very little loss of power.

**Table 1:** Power (α=0.05) for the SKAT test under the population stratification scenario and varying levels of stratification. 10 causal variants were selected in causal genes. n_2_: number of individuals of African descent out of 2000 individuals

*OR*	*n*_*2*_ *= 0*	*100*	*200*	*400*
*Power (no covariate adjustment)*				
*1.5*	*65.95%*	*58.40%*	*50.80%*	*30.77%*
*1.8*	*87.76%*	*83.35%*	*77.40%*	*54.17%*
*3*	*99.32%*	*99.25%*	*98.69%*	*93.91%*
*5*	*99.85%*	*99.90%*	*99.81%*	*99.40%*
*Power (with population as a covariate)*				
*1.5*		*64.29%*	*64.56%*	*62.52%*
*1.8*		*87.35%*	*86.77%*	*85.10%*
*3*		*99.27%*	*99.15%*	*99.25%*
*5*		*99.83%*	*99.86%*	*99.90%*

### Power calculation for rare variant (RV) association test

To assess the performance of genetic association tests with RVs under a case-control design, simulations of RV genotypes from cases and controls are necessary. *Sim1000G* can be used in this context.

As an illustration, we evaluated the statistical power of several RV association tests (Burden, SKAT and SKAT-O [18]) under different scenarios where we varied the sample size and length of the chromosomal region. The parameters for generating the data with *sim1000G* included: (a) The length *n* of the simulated region, where *n* corresponds to the number of variants with genetic variations; (b) The range of MAFs for the simulated variants.

We simulated the phenotype data of individuals using a logistic regression model similar to eq. (1). We assumed that the effect size of a causal variant is inversely proportional to its MAF, β = (ln5/4)*|log_10_MAF| [17] and that the proportion of causal variants was similar across different simulated regions. The number of cases and controls was either 250 or 1000 in each simulated dataset.

To assess the quality of the generated data, we compared *sim1000G* to the *simuRareVaraints* (SRV) script [19], implemented in the *simuPOP* [7] software, in terms of MAF distribution and association results.

We first used SRV to simulate a population of DNA sequences with RVs forward in time, subject to mutation, natural selection and population expansion. The region lengths were 50 k bp, 100 k bp and 300 k bp, which correspond to 72, 147 and 442 genetic variants respectively, in the region having mutations in the population. A gamma distribution was used for the selection coefficient of the new mutants, assuming a complex bottleneck model for the European population [20]. All other parameters of the simulator were based on the default setting of the script.

In Table 2, we notice that a larger proportion of genetic variants falls into the correct pre-specified range of MAFs when using *sim1000G* vs. SRV. In Table 3, we found that the power of the RV association tests differs depending upon which simulator was used to simulate the data. In terms of computation time, *sim1000G* was more efficient and easier to implement than *simuPOP.* Indeed, the former only simulates genotype data for a pre-specified sample size while the latter requires for each simulation scenario to generate of a huge initial population from which the final set of individuals is extracted.

**Table 2:** Proportion of variants within each MAF range category. The MAF range we specified when simulating the data was [0.0005,0.01]

		N_cases = N_controls = 250			N_cases = N_controls = 1000		
*Simulator*	*MAF range*	[0,0.0005)	[0.0005,0.01]	(0.01,0.5)	[0,0.0005)	[0.0005,0.01]	(0.01,0.5)
sim1000G	*n* = 72	9.00%	89.10%	1.80%	0.10%	98.60%	1.30%
	*n* = 147	14.30%	85.10%	0.60%	0.70%	99.10%	0.20%
	*n* = 442	14.40%	84.00%	1.60%	0.70%	98.50%	0.80%
simuPOP	*n* = 72	33.20%	65.30%	1.50%	14.60%	84.30%	1.10%
	*n* = 147	27.90%	70.00%	2.10%	11.40%	86.70%	1.90%
	*n* = 442	28.20%	69.20%	2.70%	11.70%	86.80%	1.60%

**Table 3:** Statistical power comparison

		N_cases = N_controls = 250			N_cases = N_controls = 1000		
*Simulator*	*MAF range*	SKAT	Burden	SKAT-O	SKAT	Burden	SKAT-O
sim1000G	*n* = 72	21.00%	19.00%	27.00%	70.00%	53.00%	76.00%
	*n* = 147	19.00%	32.00%	28.00%	71.00%	77.00%	82.00%
	*n* = 442	47.00%	81.00%	82.00%	98.00%	99.00%	100.00%
simuPOP	*n* = 72	26.20%	20.90%	28.80%	87.90%	59.90%	88.20%
	*n* = 147	28.70%	20.80%	30.50%	92.40%	56.30%	91.70%
	*n* = 442	66.80%	69.90%	78.40%	100.00%	99.70%	100.00%

### Family-based association test for diseases with variable age at onset

Family-based study designs allow the characterization of gene mutation effect on the disease risk by considering related individuals. A few methods have been developed for testing sets of genetic variants in family studies but only few approaches were proposed in the context of right-censored time-to-event data [21].

A correlated frailty model can be used to test the association between a set of genetic variants and a survival outcome in family studies [22]. In this model, the within-familial correlation is specified by an IBD sharing probabilities matrix. For an individual *i*, *i* = 1, … , *n*_*f*_ from a family *f* = 1, … , *n*, the risk for developing a disease is defined by the hazard function:

2 $$ {\displaystyle \begin{array}{c}{\lambda}_{fi}\left(t\left|b\right.\right)={\lambda}_0(t)\exp \left({b}_{fi}+{X}_{fi}^{\hbox{'}}\beta \right),\\ {}\mathrm{with}\kern0.5em b=\left\{{b}_{fi},i=1,\dots, {n}_f,f=1,\dots, n\right\}\sim MVN\left(0,\Sigma \left(\sigma \right)\right)\end{array}} $$

and where *λ*_0_(*t*) is a baseline hazard function, e.g. the Weibull hazard function ${\lambda}_0(t)=\frac{\rho }{\lambda }{\left(\frac{t}{\lambda}\right)}^{\rho -1}$, ${X}_{fi}^{\prime }$ is the vector of non-genetic covariates and *β* are the corresponding regression coefficients. The random effects *b* are normally distributed and correlated with each other. The covariance matrix Σ(*σ*) defines the dependence structure using the IBD matrix *B*, Σ(*σ*) = *σ*^2^*B*. The frailty parameter *σ*^2^ represents the familial correlation related to the SNP or region of SNPs included in the calculations of the IBD probabilities. The estimators of the model parameters (*ρ*, *λ*, *β*, *σ*^2^) are found using a conditional maximum likelihood estimation that accounts for the selection bias coming from the sampling of families through affected probands [23]. The procedure was implemented in the *R* package *frailtypack* [24].

We performed simulation studies where the goal was to evaluate the type I error and the power of genetic association tests when the model is correctly specified. The number of families was *N* = 100, *N* = 200 and *N* = 500. Using *sim1000G*, we generated families of three generations: parents, one or two children in the second generation and one or two children in the third generation (for each second generation individual). For generating the genotypes, we used the region from 1000 genomes Phase III sequencing data VCF files and a genetic map GRCh37 from the corresponding chromosome 4. The assumed MAF ranged from 0.02 to 0.1. The pedigree structures and mean IBD (mIBD = IBD1 + IBD2/2) were used to generate time-to-event data with the function *simfam* from the *R* package *FamEvent* [25]. We used gender as the non-genetic covariate and fixed *β*_*sex*_ to 0.5. The time of right-censoring was equal to an individual’s current age sampled from the normal distribution with variance 2.5 and mean fixed to 95, 75 and 55 for the first, second and third generation, respectively. Values for the Weibull parameters were chosen to obtain around 60% of censored cases in the samples (*λ* = 143 and *ρ* = 3.0).

For generating the survival times, we used the model (2) with genotypes as covariates to modify the risk of disease. The number of variants was *s* = 3, we assumed that they have equal effect on the disease risk, *β*_1_ = *β*_2_ = *β*_3_, this effect was fixed at 0.0, 0.5 or 1.0. In all the settings, we used model (2) for the estimation with the function *frailtyPenal* from the package *frailtypack* [20]. In order to evaluate the association test for the assumed genotypes, the frailty variance parameter, *σ*^2^, was fixed at 0.0 under the null hypothesis (no association) and estimated under the alternative (association). The *p*-values were obtained using likelihood ratio test, in which, under the null the asymptotic distribution of the test statistic is the mixture of ${\chi}_0^2$ and ${\chi}_1^2$ with equal probability 0.5.

The results of the simulation studies using 500 replicates are presented in Table 4. In all scenarios, i.e. for different number of families, the type I error was close to the nominal value of 5%. As expected, the power increased with the number of families and the assumed values of the genotypes effects *β*_*s*_. The power is low when the effect of genotypes is fixed to 0.5, which means that the test detects the genetic association only if the effect of genotypes on survival is strong. When the genotype effects are fixed to 1.0, for all sample sizes, the association test is very powerful. For datasets with 500 families, the test detects the association in all the replicates.

**Table 4:** Estimated type I error and power over 500 simulations for the association test

	*N* = 100	*N* = 200	*N* = 500
s	3	3	3
*β*_1_=⋯=*β*_s_=0	5.6	4.2	5.0
*β*_1_=⋯=*β*_s_=0.5	35.7	53.2	60.0
*β*_1_=⋯=*β*_s_=1.0	96.8	99.4	100.0

## Conclusion

We have developed the *R* package *sim1000G* for easy generation of simulated genetic variants under realistic scenarios, mimicking the 1000 Genomes project or any other phased variant call VCF files. The capabilities of the package allow the simulation of genetic variants data in tens of thousands of individuals, generated either independently or within pedigrees of arbitrary sizes.

Compared to other simulators designed to simulate existing genetic variants, *sim1000G* provides a very efficient and compelling approach to simulation, completely integrated within the *R* environment. Besides, it avoids the need for complex scripts and is independent from external packages/softwares. It allows the generation of realistic genetic variants data, from 50 variants in European families to > 1000 variants in populations of independent individuals with mixed ethnicities. S*im1000G* is able to perform these tasks under minimal computational burden, user interaction or set-up time. We have not yet implemented phenotype simulations as part of *sim1000G* to give more flexibility to the users to perform this task. A number of examples of phenotype simulations are however included in the Additional file 1.

The applications described in this paper demonstrate the versatility of *sim1000G* to perform analyses and simulations for various genetic problems. Our first application simulated unrelated individuals from a single population to compare the MAF distributions and patterns of LD of *sim1000G* to competing simulators. Our second example showed that *sim1000G* can be used to create admixed populations of unrelated individuals and such simulated data can be useful to assess methods that correct for population stratification, using either rare or common variants (or both). The third example, which simulated unrelated individuals from a single population, shows the interest of *sim1000G* to assess the power and type I error of various methods to detect genetic association with a set of RVs. Finally, our last application shows how *sim1000G* can be used to simulate sequence variants data in pedigrees and assess the power and type I error of various methods to detect genetic association in this setting.

Therefore, our implementation of *sim1000G* should facilitate future applications and developments of computational methods for association tests with both rare and common variants, using either unrelated individuals or families. Many more simulation situations than those presented here, could benefit from a package such as *sim1000G.*

Sim1000G is available for download on CRAN under the package name *sim1000G* and the most recent version is also available on github, at: https://github.com/adimitromanolakis/sim1000G.

## Availability and requirements

**Project name:** sim1000G.

**Project home page:** https://github.com/adimitromanolakis/sim1000G

**Operating system(s):** Platform independent.

**Programming language:** R.

**Other requirements:** R packages of stringr, readr and hapsim (available in CRAN).

**License:** GNU GPL.

**Any restrictions to use by non-academics:** No restrictions.

**Supplementary information:** Available on the journal’s online website.

## Additional file

Additional file 1: Supplementary materials. (PDF 216 kb)

## Abbreviations

CNV: Copy Number Variation
IBD: Identity by descent
LD: Linkage disequilibrium
MAF: Minor allele frequencies
MNP: Multi Nucleotide Polymorphism
NGS: Next-generation sequencing
NIH: National Institutes of Health
RV: Rare variant
VCF: Variant Call Format
