# Supplementary material for: sim1000G: a user-friendly genetic variant simulator in R for unrelated individuals and family-based designs
Source: BMC Bioinformatics. 2019 Jan 15;20:26. doi: 10.1186/s12859-019-2611-1 (PMC6332552; doi:10.1186/s12859-019-2611-1)
Supplement: Supplementary file 1 — Supplementary materials. (PDF 216 kb) [file 12859_2019_2611_MOESM1_ESM.pdf]

Supplementary data

1. Age of onset distribution for the simulation scenarios presented in section 4.

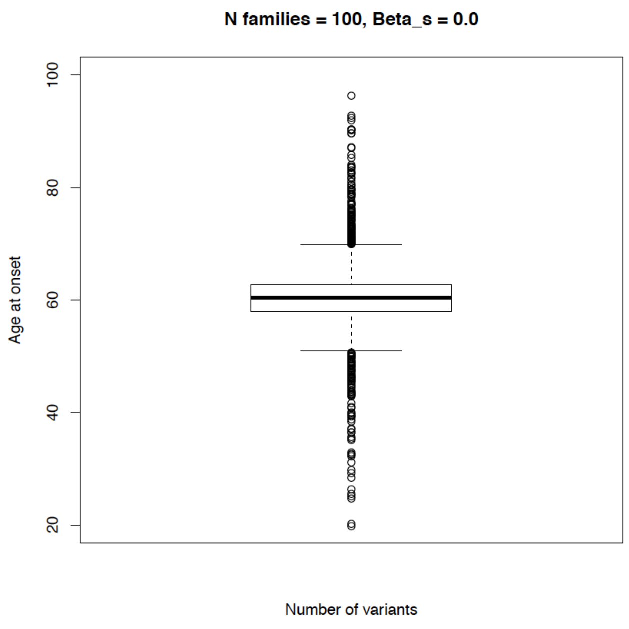

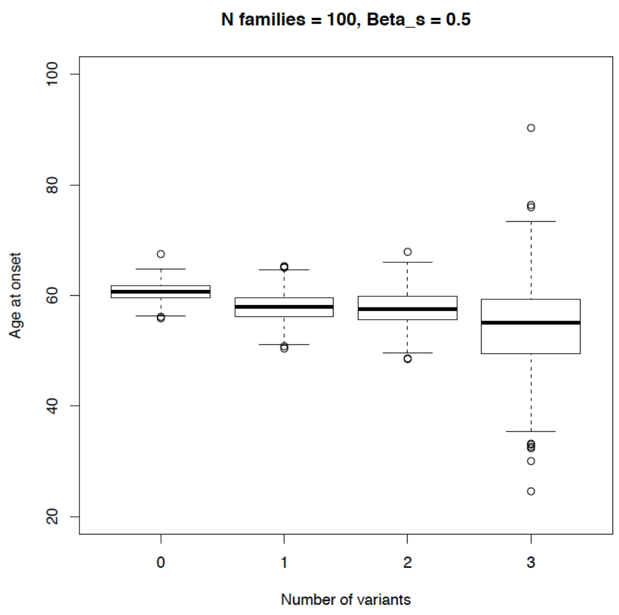

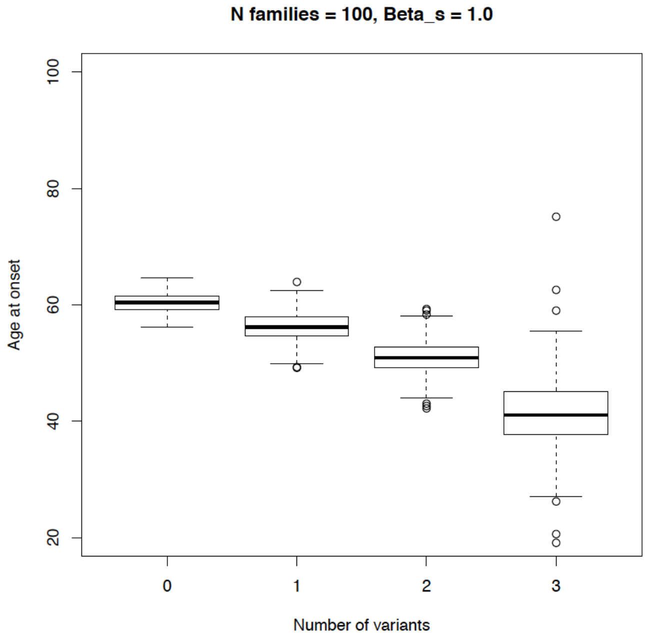

2. Comparison to hapgen2 and simugwas

Below we describe the process and simulator scripts used in the Results Section 1 of the manuscript (comparison to other simulator programs).

For hapgen2 we converted the input VCF of sim1000g to the hapgen format so the same set of haplotypes were used for simulation. Hapgen2 (version 2.1.2) was downloaded from http://mathgen.stats.ox.ac.uk/genetics_software/hapgen/hapgen2.html

The command to run hapgen2 was:

```
../hapgen2 -m genetic_map_chr1_combined_b36.txt -l  init.legend  -h init.hap  -o ex.out -dl 6185941 1 1.5 2.25   -n 2000 100 -t ./example/ex.tags -int 0 10000000
```

For sim1000G we used the following script:

```
vcf = readVCF( vcf_file, maxNumberOfVariants = 39400 , min_maf = -1)
startSimulation(vcf, totalNumberOfIndividuals = 2000)
ids <- generateUnrelatedIndividuals(2000)
```

For simuGWAS, we used the example1.py script file with default parameters but selecting the HapMap3 CEU population, chromosome 1 and region 1-10Mb. The expanded population size was 50000. The script file used was downloaded from:

```
https://github.com/BoPeng/simuPOP-examples/blob/master/published/simuGWAS/example1.py
```

3. Simulation codes for the sim1000G examples

In this document the simulation code[1] for generating the data for examples 1-4 is provided. The most recent and complete set of source code files, including the post-processing steps and additional examples, can be found in the inst/examples folder of the sim1000G package at **https://github.com/adimitromanolakis/sim1000G**.

Some example require the use of large vcf files. These vcf files are available at:

https://github.com/adimitromanolakis/sim1000G-vcf-files

[1] Code was formatted for use in a word document using the functionality in the website http://www.planetb.ca/syntax-highlight-word

EXAMPLE 1 – BASIC USAGE AND GENERATION GENERATING GENOTYPE DATA FOR A SINGLE POPULATION

```
# Example 1 , read a region from 1000 genomes, simulate 300 individuals, compute and
display LD patterns

library(sim1000G)
library(gplots)


# Read the example file included in sim1000G

examples_dir = system.file("examples", package = "sim1000G")
vcf_file = sprintf("%s/region.vcf.gz", examples_dir)

# Alternatively provide a vcf file here:
#vcf_file = "~/fs/tmp/sim4/pop1/region-chr4-312-GABRB1.vcf.gz"

vcf = readVCF( vcf_file, maxNumberOfVariants = 200 , min_maf = 0.15 ,max_maf = NA)


startSimulation(vcf, totalNumberOfIndividuals = 8000)
ids = generateUnrelatedIndividuals(20)

genotype = retrieveGenotypes(ids)

rownames(genotype) = sprintf("individual %d",1:nrow(genotype))

heatmap(genotype,col=c("white","orange","red"), Rowv=F)
```

EXAMPLE 2 – EVALUATING POPULATION STRUCTURE CORRECTED USING GENOTYPES FROM AN ETHNIC MIXTURE OF POPULATIONS

```
library(sim1000G)
library(parallel)
```

```
library(SKAT)

par = commandArgs(T)

NREP = 15  # How many replicates to run for each case

#### Initialize the simulation ####


# List all available gene region vcf files,
# the vcf files should be located in two directories called pop1 and pop2

all_vcf_files = list.files(path="pop1",  full.names = T)
selected_vcf_file = sample(all_vcf_files , 1)


# Find corresponding vcf file of second population
selected_vcf_file2 = gsub("pop1","pop2", selected_vcf_file)



cat("Reading the two vcf files: " , selected_vcf_file,selected_vcf_file2 ,"\n")




initSimulation = function() {


    ## Read two VCF file with regions from two different populations
    ## vcf1: variants from european population
    ## vcf2: variants from african populations


    gn = strsplit(selected_vcf_file,"-")
    gn = sapply(gn, function(x) x[4])

    genename <<- gn
    geneid = gn

    vcf1 <<- readVCF(selected_vcf_file ,
                    maxNumberOfVariants = 800,  min_maf =  1e-6, max_maf = 0.02)


    if(class(vcf1) != "environment") stop(1);


    vcf2 <<- readVCF( selected_vcf_file2  ,
                    maxNumberOfVariants = 800,  min_maf = 1e-6, max_maf = 0.02)


    print(class(vcf1))
    print(class(vcf2))


    if(class(vcf1) != "environment") stop(1);
    if(class(vcf2) != "environment") stop(1);




    ## We select only the common variants between the 2 VCF files
    ##

    common = intersect(vcf1$varid,vcf2$varid)
    print(length(common))

    if(length(common) < 10) stop("less than 10 common variants, use vcf files with more
```

```
variants");

    vcf1 <<- subsetVCF(vcf1, var_id = common)
    vcf2 <<- subsetVCF(vcf2, var_id = common)

    cat(geneid, length(common)," \n");


    common_causal_pool <<- 1:length(vcf1$maf)

    cat("start sim\n");


    startSimulation(vcf1, totalNumberOfIndividuals = 10000)
    saveSimulation("pop1")

    startSimulation(vcf2, totalNumberOfIndividuals = 10000)
    saveSimulation("pop2")


    print(length(vcf1$varid))

}





SEED = 1

#### Function to generate one simulation replicate ####




predictor_logistic = function(b, geno) {
    x = b[1]
    for(i in 1:ncol(geno)) { x = x  + b[i+1] * ( geno[,i] > 0) + b[i+1] * ( geno[,i] > 1)
}
    exp(x) / (1+exp(x) )
}





replicate1 = function(seed=NA,
                     numcausal = 10,
                     effect_size = 10,
                     pop_strat = 0,
                     N_pop1 = 400,
                     N_pop2 = 200
                    )
{


        loadSimulation("pop1")

        id = generateUnrelatedIndividuals(N_pop1)

        genotypes = retrieveGenotypes(id)
        rownames(genotypes)= rep(  "CEU" , nrow(genotypes) )


        genotypes2 = NA
        gt = genotypes

        if(N_pop2 > 0) {
```

```
        loadSimulation("pop2")
        id = generateUnrelatedIndividuals(N_pop2)


        genotypes2 = retrieveGenotypes(id)
        rownames(genotypes2)= rep(  "ASW" , nrow(genotypes2) )

        gt = rbind(genotypes,genotypes2)
    }



    pca = prcomp(gt)


    maf = apply(gt,2,mean,na.rm=T)/2
    apply(gt,2,function(x) sum(is.na(x)))
    flip  = which(maf > 0.5) ; gt[,flip] = 2 - gt[,flip]
    maf = apply(gt,2,mean,na.rm=T)/2



    maf_gt0 = which(maf > 0)

    effect_sizes = rep(0, ncol(gt))
    nvar = length(effect_sizes)

    s = sample(common_causal_pool, numcausal)
    effect_sizes[s] = effect_size




    S=0

    while(1) {
            p = rep(NA, nrow(gt) )

            s = rownames(gt) != "ASW"

            p[s] = predictor_logistic (  c(S,effect_sizes) ,  gt[s,])
            p[!s] = predictor_logistic (  c(S+pop_strat,effect_sizes) ,  gt[!s,])

            phenotype = rbinom( length(p) , 1 , p )


            ncases = sum(phenotype==0)
            ncontrols = sum(phenotype==1)

            # cat(ncases,ncontrols,"\n")
            if( abs(ncases-ncontrols) < 5 ) break();

            if(ncases > ncontrols) S = S + 0.1
            if(ncases < ncontrols) S = S - 0.1
    }



    t=( table(rownames(gt)  , phenotype ) )
    print(t)




    #phenotype = sample(phenotype)

    obj<-SKAT_Null_Model(phenotype ~ 1, out_type="D")
```

```
        pv = SKAT((gt),obj)$p.value



        if(sum(rownames(gt) != "CEU") > 0) {

            population = factor(rownames(gt))

            obj<-SKAT_Null_Model(phenotype ~ population, out_type="D")

            # obj<-SKAT_Null_Model(phenotype ~
            #      pca$x[,1] + pca$x[,2] + pca$x[,3] + pca$x[,4] + pca$x[,5] + pca$x[,6],
out_type="D")

            pv_cov = SKAT((gt),obj)$p.value


        } else {
            pv_cov = -1
        }



        cat("EFF= ", effect_size,
            "POP1=",N_pop1,
            " POP_STRAT=" , pop_strat,
            " SKATPV=", pv,
            " SKATPV_COV=", pv_cov,
            " GENE=", genename,
            # " t1= ", table(rownames(gt)  , phenotype  ),

            "\n");


        tbl = table(rownames(gt),phenotype)
        data.frame(ID="EFFZZ", eff=effect_size,strat=pop_strat,pop1=N_pop1, pop2=N_pop2,
                   pv1=pv,pv2=pv_cov,
                   gene=genename

                   )
}


#### Generate multiple simulation replicates ####

rand_name = function() {

    x = sprintf("%04d",round(runif(10,1,1000)) )
    paste(x,sep="",collapse="")

}




base_seed = round( runif(1,0,1e9) )

initSimulation()





clusterRep = function(x) {

    replicate1(seed=base_seed + 101*x, numcausal = 10, effect_size = EFF,
               N_pop1 = 2000-p2,
```

```
                N_pop2 = p2,
                pop_strat = pop_strat
    )
}


results = list()


pop_strat_list = c(2)
effect_sizes = c(0,log(1.5), log(1.8) , log(3), log(5)  )

for(pop_strat in pop_strat_list)
    for(EFF in effect_sizes)
        for(p2 in c(0,100,200,400))

{


        cat(pop_strat, EFF, p2,"\n");

        v = lapply(1:NREP, clusterRep)

        results = c( results  ,  v   )
}





pz = plyr::ldply(results)
pz$MASTER_ID = "REPLICATE"

pz$pv2 [pz$pv2 < 0] = NA

reshape2::dcast(pz, eff + factor(pop2) ~ 1,value.var="pv1",function(x) mean(x<0.05))
reshape2::dcast(pz, eff + factor(pop2) ~ 1,value.var="pv2",function(x) mean(x<0.05))


save(results,pz,file=sprintf("sim-results-%s.rdata",rand_name()) )

write.table(pz,  row=F,quote=F,sep=" ", col=F, file=sprintf("out-%s.txt", rand_name()) )
```

EXAMPLE 3 – GENERATION OF RARE VARIANTS AND RUNNING SKAT ON A SIMULATED PHENOTYPE

```
library(sim1000G)
vcf_file = "region-chr4-357-ANK2.vcf.gz" #nvariants = 442, ss=1000


vcf = readVCF( vcf_file, maxNumberOfVariants = 442  ,min_maf = 0.0005,max_maf = 0.01)
#lowest MAF
dim( vcf$gt1 ) #rows represent number of variants, columns represent number of
individuals
```

```
## Download and use full chromosome genetic map
downloadGeneticMap(4)
readGeneticMap(4)



sample.size=3000


startSimulation(vcf, totalNumberOfIndividuals = sample.size)


data_sim = function(seed.num){

  SIM$reset()

  id = generateUnrelatedIndividuals(sample.size)

  gt = retrieveGenotypes(id)


  freq = apply(gt,2,sum)/(2*nrow(gt))
  causal = sample(setdiff(1:ncol(gt),which(freq==0)),45)

  beta.sign = rep(1,45)
  c.value = 0.402
  beta.abs = c.value*abs(log10(freq[causal]))
  beta.val = beta.sign*beta.abs
  x.bar = apply(gt[,causal],2,mean)
  x.bar = as.matrix(x.bar)
  beta.val = t(as.matrix(beta.val))
  #disease prvalance = 1%
  #beta0 = -log(99)-beta.val %*% x.bar
  #disease prvalance = 1.5%
  beta0 = 0-beta.val %*% x.bar

  eta = beta.val %*% t(gt[,causal])
  eta = as.vector(eta) + rep(beta0,nrow(gt))
  prob = exp(eta)/(1+exp(eta))

  genocase = rep(NA, sample.size)

  set.seed(seed.num)
  for(i in 1:sample.size){
    genocase[i] = rbinom(1, 1, prob[i])
  }
  case.idx = sample(which(genocase==1),1000)
  control.idx = sample(which(genocase==0),1000)

  return(rbind(gt[case.idx,],gt[control.idx,]))
}


library(SKAT)
  res = NULL

  for(seed_number in 1:100) {
    set.seed(seed_number)
    print(seed_number)

    Z1.skat = data_sim(seed_number)

    write.csv(Z1.skat,paste("data_SKAT_2000_442_",seed_number,".csv",sep=""),row.names   =
F,quote=F)

    obj = SKAT_Null_Model(c(rep(1,1000),rep(0,1000)) ~ 1,out_type="D")

    p1_skat = SKAT(as.matrix(Z1.skat),obj)$p.value
```

```
      p1_burden = SKAT(as.matrix(Z1.skat),obj,r.corr=1)$p.value
      p1_skat_O = SKAT(as.matrix(Z1.skat),obj,method="optimal.adj")$p.value

      res = rbind(res,c(p1_skat,p1_burden,p1_skat_O))
    }


   write.csv(res,"skat_442_2000.csv",row.names = F,quote=F)
```

Example 4 – Generating Family data and frailty model

```
  library(sim1000G)
  library(FamEvent)
  library(frailtypack)

  set.seed(122)
  # Initialize simulator of family data using 1000 genome haplotypes for each cluster
  vcf <- readVCF("../region.vcf.gz", maxNumberOfVariants = 100, min_maf = 0.02, max_maf =
  0.1)
  readGeneticMap(chromosome = 4)
  startSimulation(vcf, totalNumberOfIndividuals = 2000)


  # Number of families
  nfam <- 200
  # Define parameters of the model
  Beta <- c(0.5,1.0,0.5,0.3)
  ver <- length(Beta)
  sig2 <- 0.7
  Weibull <- c(0.007,3)
  variation <- "IBD"


    # Simulate families
    SIM$reset()
    ## For simulations, we set the cM that the regions spans to 4000 to diversify the IBD
  matrix
    ## Remove for normal use
    SIM$cm = seq( 0,4000, length = length(SIM$cm) )

    # Generate family pedigries and their genotypes
    time_x_families <- function(){
      fam  <-  lapply(paste(1:nfam),  function(x){  noffspring2  <-  sample(c(1:2),  1,
  replace=TRUE,prob=c(0.5, 0.5))#, 0.1482
      noffspring3 = sample(c(1,2), noffspring2, replace=TRUE,prob=c(0.8518, 0.1482))#rep(1,
  noffspring2)#
      newFamily3generations(x,  noffspring2 = noffspring2, noffspring3)})
      fam <- do.call(rbind, fam)

      fam
    }

    fam <- time_x_families()

    # Transform the data to have ids from 1 to n, n - number of individuals
    nmemb <-as.vector(table(fam$fid))
    cumsum_nmemb <- c(0,cumsum(nmemb))
    fam$ID <- 1:dim(fam)[1]
    fam$fatherID <- 0
    fam$fatherID              <-              sapply(1:dim(fam)[1],              function(x)
  fam$ID[as.numeric(fam$father[which(fam$ID==x)])+cumsum_nmemb[fam$fid[x]]])
```

```
  fam$fatherID <- ifelse(fam$father==0, 0, fam$fatherID)
  fam$fatherID <- as.numeric(fam$fatherID)
  fam$motherID <- 0
  fam$motherID              <-              sapply(1:dim(fam)[1],              function(x)
fam$ID[as.numeric(fam$mother[which(fam$ID==x)])+cumsum_nmemb[fam$fid[x]]])
  fam$motherID <- ifelse(fam$mother==0, 0, fam$motherID)
  fam$motherID <- as.numeric(fam$motherID)
  fam <- fam[,c(1,8:10,5:7)]
  colnames(fam)  <-  c("famID",  "ID",  "fatherID",  "motherID",  "gender",  "generation",
"gtindex")



  # Create data of genotypes
  genotype <- rep(0,length(SIM$gt1[1,]))
  for(i in  1:dim(fam)[1]){
    gen                                                                                  <-
paste(SIM$gt1[as.numeric(fam$gtindex[i]),],SIM$gt2[as.numeric(fam$gtindex[i]),],   sep   =
"")
    gen <- ifelse(gen == "00", 3, gen)
    gen <- ifelse(gen%in%c("10","01"), 2, gen)
    gen <- ifelse(gen == "11", 1, gen)
    genotype <- rbind(genotype, as.numeric(gen))
  }

  genotype <- genotype[-1,]
  colnames(genotype) <- paste("Gen", 1:dim(genotype)[2], sep="")

  # Create mean IBD matrix
  n = SIM$individuals_generated
  vec <- rep(0, length(nmemb))
  vec2 <- vec
  vec2[2:length(vec)] <- vec[2:length(vec)] + nmemb[-length(nmemb)]
  vec3 <- cumsum(vec2)+1
  nmemb2 <- cumsum(nmemb)
  IBD2matrix <- matrix(rep(0,n^2), nrow = n)

  for(i in 1:length(nmemb)){
    IBD2matrix[vec3[i]:nmemb2[i],vec3[i]:nmemb2[i]]      <-      sapply(vec3[i]:nmemb2[i],
function(y) {
      z = sapply(vec3[i]:nmemb2[i], function(x) computePairIBD12(x,y))
      # names(z) = 1:n
      z
    })
  }
  colnames(IBD2matrix) = 1:nrow(IBD2matrix);rownames(IBD2matrix) = 1:nrow(IBD2matrix)

  # Generate time-to-event data using simfam function from the package FamEvent
  fam_FamEvent <- simfam(N.fam = length(unique(fam$famID)), design = "pop+", variation =
variation,
                           base.dist  =  "Weibull",  frailty.dist  =  "lognormal",  depend  =
sig2,
                           base.parms = Weibull, vbeta = Beta,
                           agemin = 0, data.fam = fam, genotype = NULL, IBD = IBD2matrix,
                           age1 = c(95, 2.5), age2 = c(75, 2))

  fam_FamEvent$gender2 <- ifelse(fam_FamEvent$gender == 0, 2, fam_FamEvent$gender)
  family <- fam_FamEvent[order(fam_FamEvent$indID),]

  family$t0  <-  0  #  for  frailtypack,  we  create  dummy  variable  0  for  starting  time  of
observation

  # Fit the model using function frailtyPenal from frailtypack package
  fit  <-  frailtyPenal(Surv(t0,  time,  status)  ~  gender  +cluster(famID),  data  =  family,
hazard="Weibull",
                      RandDist   =   "LogN",   print.times   =   FALSE,   init.B   =   Beta[1],
covMatrix1 = IBD2matrix,
                      recurrentAG = TRUE, maxit = 35,
```

```
                          proband = family$proband, currentage = family$currentage)


    #Genotypes included in generating time-to-event data
      fam_FamEvent <- simfam(N.fam = length(unique(fam$famID)), design = "pop+", variation
  = variation,
                               base.dist = "Weibull", frailty.dist = "lognormal", depend =
  sig2,
                               base.parms = Weibull, vbeta = Beta,
                               agemin = 0, data.fam = fam, genotype = genotype[,1:3], IBD =
  IBD2matrix,
                               age1 = c(95, 2.5), age2 = c(75, 2))


    fam_FamEvent$gender2 <- ifelse(fam_FamEvent$gender == 0, 2, fam_FamEvent$gender)
    family <- fam_FamEvent[order(fam_FamEvent$indID),]

    family$t0 <- 0 # for frailtypack, we create dummy variable 0 for starting time of
  observation

    # Fit the model using function frailtyPenal from frailtypack package
    fit2 <- frailtyPenal(Surv(t0, time, status) ~ gender +cluster(famID), data = family,
  hazard="Weibull",
                          RandDist = "LogN", print.times = FALSE, init.B = Beta[1],
  covMatrix1 = IBD2matrix,
                          recurrentAG = TRUE, maxit = 35,
                          proband = family$proband, currentage = family$currentage)
```
